# 2015: which new directions for Alzheimer's disease?

**DOI:** 10.3389/fncel.2014.00417

**Published:** 2014-12-09

**Authors:** Xénia Latypova, Ludovic Martin

**Affiliations:** Université de NantesNantes, France

**Keywords:** Alzheimer's disease (AD), dementia, tau, abeta, therapeutics

Around 44 million people in the world are suffering from dementia including Alzheimer's disease (AD). It is considered as one of the biggest global public health challenges our generation cope with. At the dawn of 2015, AD medical care remains unsuccessful despite the identification of its neuropathological hallmarks one century ago. Being attentive to emerging prospects is essential because the current advances lead to substantive improvement of the medical care. We are pleased to present an issue specifically devoted to AD and to the related therapeutic strategies. The Topic Research in this issue is based on a series of original papers and reviews. The latters focus on the advances in basic and clinical research trends in AD, they provide an up-to-date information and future perspectives on this hot topic as well.

AD is a progressive disease, it occurs over a long period before the onset of symptoms which are impaired memory, apathy, and depression. The characteristics of AD consist in neurofibrillary tangles (intraneuronal aggregates of hyperphosphorylated tau proteins) and senile plaques [dense extraneuronal deposits composed of amyloid β (Aβ)]. The other features linked to these two core pathological hallmarks of AD are inflammation, oxidative stress, progressive synaptic, and neuronal loss. Nowadays, many AD molecular patterns have been screened to identify a potential therapeutic strategy. Although a myriad of evidence shows that the hippocampal volume decrease belongs to the AD earliest signs, as it is pointed out by the authors of the review paper presented in this issue, this element clearly could not be used as a diagnostic criterion (Maruszak and Thuret, 2014).

With the flood of evidence for tau pathology as key event of the disease development, the understanding of diverse tau functions and its molecular behavior is one of the major steps in the progression of our knowledge about the neurodegenerescence detected in AD. The precision of tau role in DNA protection and RNA integrity under physiological conditions or under ROS-producing stress (Violet et al., 2014) provides clarification for a mechanistic model in which tau disturbance initiates an explanation for DNA damages observed in AD. Principally, tau is a phosphoprotein. So, a complex equilibrium between tau kinases and phosphatases activities is one of the main potential therapeutic runways. Abnormal or excessive tau phosphorylation by either kinases such as GSK3β, CDK5, DYRK1A for example or other known and unknown kinases are related to AD pathogenesis. However, the identity and the strict number of tau kinases involved in AD process remain uncertain. In this way, focus at specific tau phosphorylation site(s) by a kinase multitargeting approach as potential AD therapeutic strategy has been proposed to effectively hamper the multifactorial disease progression (Hilgeroth and Tell, 2013). Since diabetes, linked itself to dysregulation of GSK3β activity, is associated in late-life with an increased risk of dementia, epidemiological and experimental studies are summarized in this issue in order to understand the effects of diabetes mellitus on tau pathogenesis. In fact, the authors discuss herein a link between tau, diabetes mellitus and the cognitive impairment (El Khoury et al., 2014). On the other hand, Peptidyl-prolyl cis-trans Isomerase NIMA-interacting 1 (Pin1), which plays a role in the balance of phosphorylation/dephosphorylation of tau, has been suspected of participating in a common mechanism between AD and hypoxia. The pathophysiological relevance of Hypoxia-inducible factor 1α (HIF-1α) pathway regulation in APP amyloidogenic metabolism has been explored in order to clarify the relationship between AD and hypoxia (Lonati et al., 2014). Among tau-based therapeutic strategies, the one which limits the spreading of tau pathology without affecting tau intracellular functions is especially attractive (Medina and Avila, 2014). Strong support for this idea comes from the importance of tau for Aβ-induced synaptotoxicity. In addition to this, high concentration of Aβ inhibits synaptic activity and in turn, it involves toxicity for the targeted synapses (Wang et al., 2013). According to this strategy, synaptic inhibitory systems could help to compensate neurotransmission imbalance (Nava-Mesa et al., 2014). Moreover, natural anti-inflammatory components, as Andrean Compound, has been studied as a preventive or adjuvant agent in AD (Maccioni et al., 2014).

Accumulation of Aβ is induced when the equilibrium between its production and clearance is disrupted. Thus, amyloid-based therapeutic strategies target this balance but numerous trials were unsuccessful for reasons of specificity or biodisponibility. However, the restoration of the microglial homeostasy and the targeting of Triggering Receptor Expressed on Myeloid cells 2 (TREM2) pathway can constitute an elegant therapeutic strategy (Zhao and Lukiw, 2013). Compounds are yet available to improve the microglia-dependent clearance of Aβ, and this strategy would benefit from *in vivo* experiments (Jones et al., 2014). Furthermore, diversity of gut microbiota changes through life stages and alterations of this ecosystem have been associated with diverse diseases. Therapeutic modulation of gut microbiota could constitute a promising adjuvant therapy in central neurodegenerative disorders. An opinion article proposes probiotics as a prophylactic treatment to prevent AD in its early stages (Bhattacharjee and Lukiw, 2013).

Several recent key advances in the field of AD understanding and treatment are presented in this thematic issue. Ultimately, we hope that you will enjoy as us to read these papers and reviews presented in this special issue. The coming years certainly promise a time for completion of a new era in the history of the AD understanding and emerging therapies. AD understanding is within reach. Stay tuned.

## Conflict of interest statement

The authors declare that the research was conducted in the absence of any commercial or financial relationships that could be construed as a potential conflict of interest.
